# Muscle quality index and isometric strength in older adults with hip osteoarthritis

**DOI:** 10.7717/peerj.7471

**Published:** 2019-08-07

**Authors:** Daniel Jerez-Mayorga, Luis Javier Chirosa Ríos, Alvaro Reyes, Pedro Delgado-Floody, Ramon Machado Payer, Isabel María Guisado Requena

**Affiliations:** 1 Physical Education and Sports Department, Faculty of Sport Sciences, University of Granada, Granada, Spain; 2 Faculty of Rehabilitation Sciences, Universidad Andres Bello, Santiago, Chile; 3 Department of Physical Education, Sports and Recreation, Universidad de La Frontera, Temuco, Chile; 4 Nursing, Physiotherapy and Occupational Therapy Department, Faculty Nursing of Albacete, University of Castilla-La Mancha, Albacete, Spain

**Keywords:** Muscle quality, Hip, Osteoarthritis, Strength, Isometric, Elderly

## Abstract

**Background:**

Older adults with hip osteoarthritis (OA) suffer a progressive loss of muscle quality and strength, affecting their daily activities and quality of life. The purpose of this study is to compare the levels of isometric strength among older adults with and without hip OA and healthy young adults, and to determine the relationship between muscle quality index (MQI) and isometric strength.

**Methods:**

Fourteen subjects with hip OA (65.6 ± 3.0 years), 18 healthy older adults (66.6 ± 6.5 years) and 32 young adults (20.7 ± 2.0 years) participated in the study. MQI, isometric muscle strength of the hip, ten time sit-to-stand tests, and body composition were measured.

**Results:**

The MQI was lower in subjects with hip OA, with no significant differences between groups (*p* > 0.054). Subjects with OA produced significantly less isometric strength in hip extension (*p* < 0.001), flexion (*p* < 0.001), abduction (*p* < 0.05), adduction (*p* < 0.001), external (*p* < 0.05) and internal rotation (*p* < 0.05). Subjects with OA demonstrated longer time in the execution of the sit-to-stand test (*p* < 0.001) in comparison with healthy older and young adults. High correlations between MQI, sit-to-stand (*r* =  − 0.76, *p* < 0.01) and peak force during hip abduction (*r* = 0.78, *p* < 0.01) where found in subjects with OA. Moderate correlation between MQI and peak force during hip flexion (*r* = 0.55, *p* < 0.05) and external rotation (*r* = 0.61, *p* < 0.05) were found in the OA group.

**Conclusions:**

Subjects with OA have lower MQI than old and young healthy controls. In subjects with OA, there was a significant relationship between isometric strength of hip muscles and performance on the sit-to-stand test and the MQI.

## Introduction

Hip osteoarthritis (OA) is a prevalent musculoskeletal condition that generates pain, disability, and deteriorates quality of life (Pereira et al., 2011; Zhang et al., 2005; Zhang et al., 2010). Around 11% of elderly people are affected by OA. Patients with hip OA have gait abnormalities (Diamond et al., 2018), hip muscles weakness and atrophy (Loureiro, Mills & Barrett, 2013), reduced functional capacity (Judd et al., 2014), and range of motion of the hip (Arokoski et al., 2004).

Physical activity (Rausch Osthoff et al., 2018) and specific muscle strengthening are key components of hip OA rehabilitation (Bennell & Hinman, 2011). Isometric strength of hip muscles is associated with higher physical function in patients with hip OA (Hall et al., 2017). In addition, patients with OA show reduced volume of the hip musculature, increased fat percentage and decreased strength of the hip muscles (Momose et al., 2017; Zacharias et al., 2016). Atrophy of gluteal muscles is also associated with clinical severity of hip OA (Zacharias et al., 2018).

Muscle quality (MQ) describes physiological functional capacity of muscle tissue (Fragala, Kenny & Kuchel, 2015); it includes aspects of anatomic structure, chemical composition, and metabolic and mechanical performance of the muscles (Heymsfield et al., 2015). MQ is directly associated with the strength per unit of muscle mass (Moritani & DeVries, 1979). Upper and lower extremity MQ decreases as age increases (Newman et al., 2003). MQ may provide a differential diagnosis of poor physical performance (Chiles Shaffer et al., 2017), and it may be a strong predictor of lower-extremity physical function in older men and women (Straight, Brady & Evans, 2015a; Straight, Brady & Evans, 2015b).

MQ is a determinant factor of muscle function (McGregor, Cameron-Smith & Poppitt, 2014). The loss of MQ and muscle power may underlie the impairments in motor control and balance that lead to falls (Martinikorena et al., 2016). Falls are one of the leading causes of morbidity and mortality in the adult population, and previous studies have shown that there is an increased risk of falls associated with hip OA (Doré et al., 2015; Van Schoor, Lips & Deeg, 2017).

To assess MQ in clinical and community practice, there are different methodologies that allow for a precise diagnosis, such as magnetic resonance imaging, computed tomography (CT), dual-energy X-ray absorptiometry, bioelectrical impedance analysis, and ultrasound (Heymsfield et al., 2015). Most of these methods involve excessive cost and difficulty of access for clinicians and patients; therefore, it is essential to have an easily accessible test, such as bioelectrical impedance analysis, that allows healthcare professionals to evaluate MQ in older adults.

The intramuscular changes associated with performance and sarcopenia during aging have led to the development of MQ measurement strategies in this type of population (Barbat-Artigas et al., 2012; Fragala et al., 2014; Fragala, Kenny & Kuchel, 2015). Performance-based assessments of muscle power via timed tests of function and body size estimates associated with lower extremity muscle strength may be responsive to age-related changes in MQ (Correa-de Araujo et al., 2017). Takai et al. (2009) developed the muscle quality index (MQI) as a tool to assess lower limb muscle power in older adults. The MQI includes anthropometric measures, such as body mass and length of the lower extremity, and the time in the sit-to-stand test. This test is an indicator of functional independence in older adults (Van Lummel et al., 2016). The ability to transfer from the sitting position to the standing position is a prerequisite to functional independence, hence the importance of being evaluated (Van Lummel et al., 2016). On the other hand, a low MQI has been correlated with a higher probability of dying compared to those witha higher MQI (Brown, Harhay & Harhay, 2016). Also, isometric exercise and training would improve muscle morphology and rapid force production and induce analgesia (Oranchuk et al., 2019; Rio et al., 2017).

In this context, there is no evidence for the use of MQI in elderly subjects affected by some degenerative pathology; in turn, it is not clear what relationship exists between isometric hip strength and anthropometric variables between subjects with OA, older adults without OA and healthy young people.

The purpose of this study was to compare the levels of isometric strength among older adults with OA and without hip OA and healthy young adults, and to determine the relationship between MQI and isometric strength. We hypothesized that: (I) subjects with OA present a lower MQI and lower isometric hip strength compared to older adults without hip OA or healthy young adults; (II) there is a high relationship between levels of isometric strength of the muscles acting in the hip joint and MQI in older adults with OA.

## Method

### Participants

For this cross-sectional study, 14 subjects with hip OA (65.6 ± 3.0 years), 18 healthy older adults (66.6 ± 6.5 years), and 32 young adults (20.7 ± 2.0 years) were recruited. Subjects with unilateral diagnoses of hip OA who met the inclusion criteria were identified by a specialist OA doctor at a local hospital. Radiographic hip OA was defined using Kellgren & Lawrence’s (1957) radiographic severity grade of ≥2. Subjects who had not reported any other orthopedic comorbidity, reconstructive joint replacement, neuromuscular diseases, neurological complications, or back pain that could affect gait were recruited for the study.

Other inclusion criteria included older adults without hip OA and young adults with no cardiovascular, lung, or metabolic pathologies and no radiological evidence of hip OA, and participants with no reported musculoskeletal pain within the three months before baseline assessments. The study protocol followed the Helsinki Declaration of 2013, and the study was approved by the Scientific Ethics Committee of the University of Granada, Spain (No 619/CEIH/2018). Each patient and participant was informed about the purpose and procedures of this study and all possible risks during measurements. All participants provided written informed consent before taking part in the study. Anthropometric characteristics of the participants are provided in Table 1.

**Table 1:** Descriptive characteristics of study participants.

Variable	OA Mean (SD) (*n* = 14)	HYA Mean (SD) (*n* = 32)	HOA Mean (SD) (*n* = 18)
Age (years)	65.6 (3.0)	20.7 (2.0)	66.6 (6.5)
Body mass (kg)	76.3 (10.7)	59.0 (8.8)	67.8 (11.2)
Height (cm)	155.2 (8.9)	163. 3 (6.6)	162.2 (4.3)
BMI (kg/m2)	31.7 (4.3)	21.9 (2.1)	25.6 (3.6)
Lean Mass (kg)	44.2 (10.0)	48.6 (9.3)	43.3 (5.5)
Body Fat (%)	24.2 (6.2)	11.5 (5.3)	24.4 (8.4)
Leg Length (m)	0.76 (0.03)	0.79 (0.04)	0.81 (0.04)

**Notes.**

MQI Muscle Quality Index OA Osteoarthritis HYA healthy young adult HOA healthy older adult SD Standard deviation

### Anthropometric measurements

Anthropometric measures were determined using a calibrated balance and a graduated stadiometer (SECA^®^). Body mass index (BMI) was calculated using the following formula: BMI = kg/m^2^. Leg length was measured manually, applying the anthropometric measurement protocol of an internationally validated recommendations society (Stewart, Marfell-Jones & Olds, 2011). A measuring tape was used for this assessment. Leg length was defined as the distance (in meters) from the greater trochanter of the femur to the lateral malleolus. A Tanita SC-330 body composition analyzer (Tanita, Tokyo, Japan) was used to measure muscle and fat mass (Jebb et al., 2000).

### Muscle strength evaluation

Maximum voluntary isometric contraction of the hip joint muscles was measured using a functional electromechanical dynamometer (DEMF) on the non-dominant leg. The DEMF allows isometric assessment of muscle strength (5-3000N) with a sampling frequency of 1,000 Hz (Dynasystem, Model Research, Granada, Spain) (Chamorro et al., 2018; Vega et al., 2018). The peak of maximum force (PF) expressed in newtons was used for further analysis.

Before testing, each subject performed an adequate warm-up, consisting of two to three sub-maximal contractions of the hip muscles to become familiar with testing procedures. Each subject performed a maximum voluntary isometric contraction for 6 s, three times, with at least one minute of rest between the trials to avoid fatigue. Flexion, extension, abduction, adduction, internal and external rotation movements of the hip were tested.

### Hip flexion

**Figure 1:**
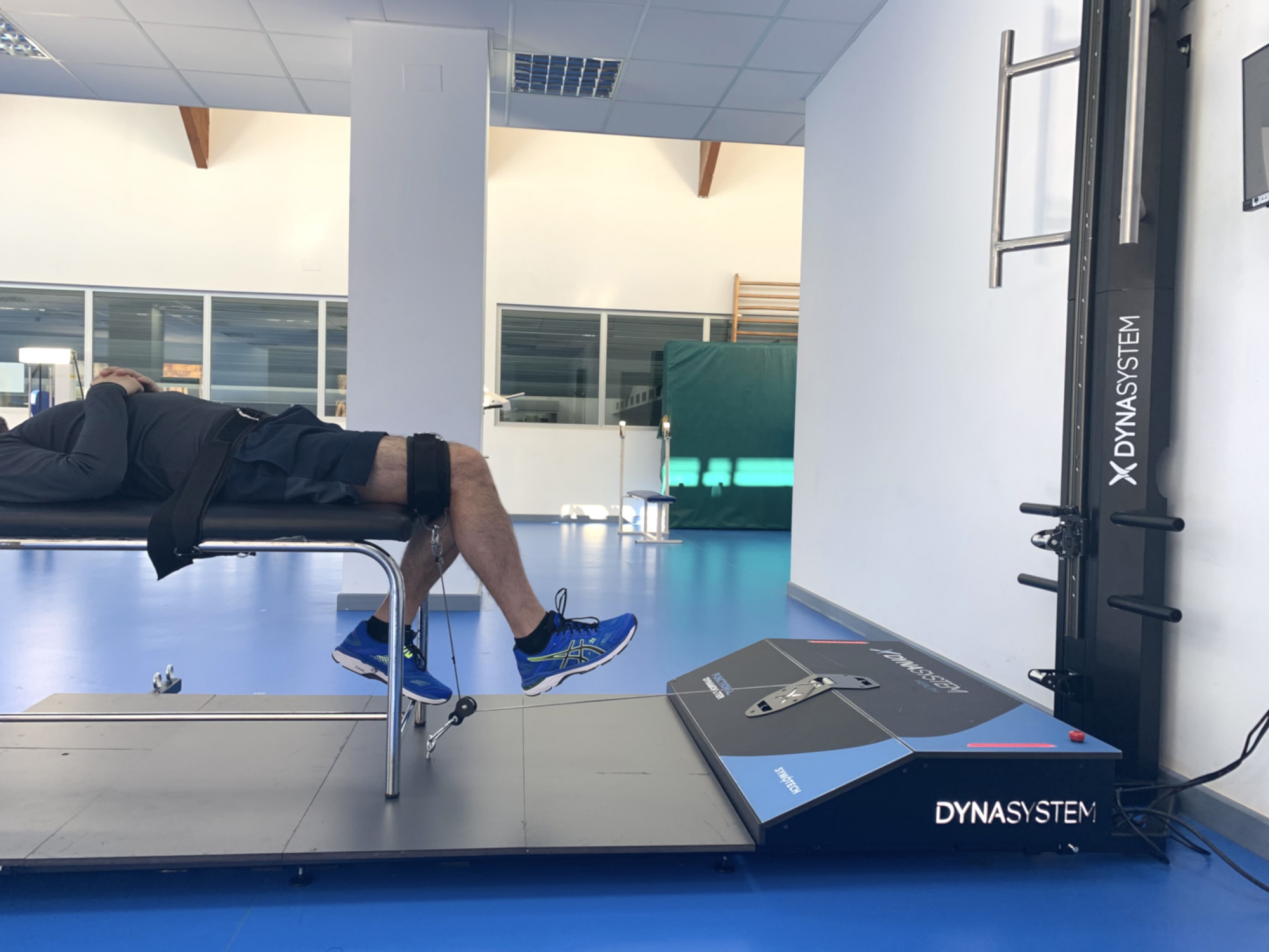
Hip flexion. Isometric hip flexion using a functional electromechanical dynamometer.

Hip flexion was evaluated as follows: the subject was placed in a supine position, with the knees on the outside of the stretcher forming an angle of 90°. The hip was stabilized with a belt around the stretcher. The axis of the hip joint was aligned with the axis of the DEMF pull. The subject was instructed to perform a hip flexion muscle contraction as strong as possible against a fixed resistance provided by the DEMF (Fig. 1).

### Hip extension

**Figure 2:**
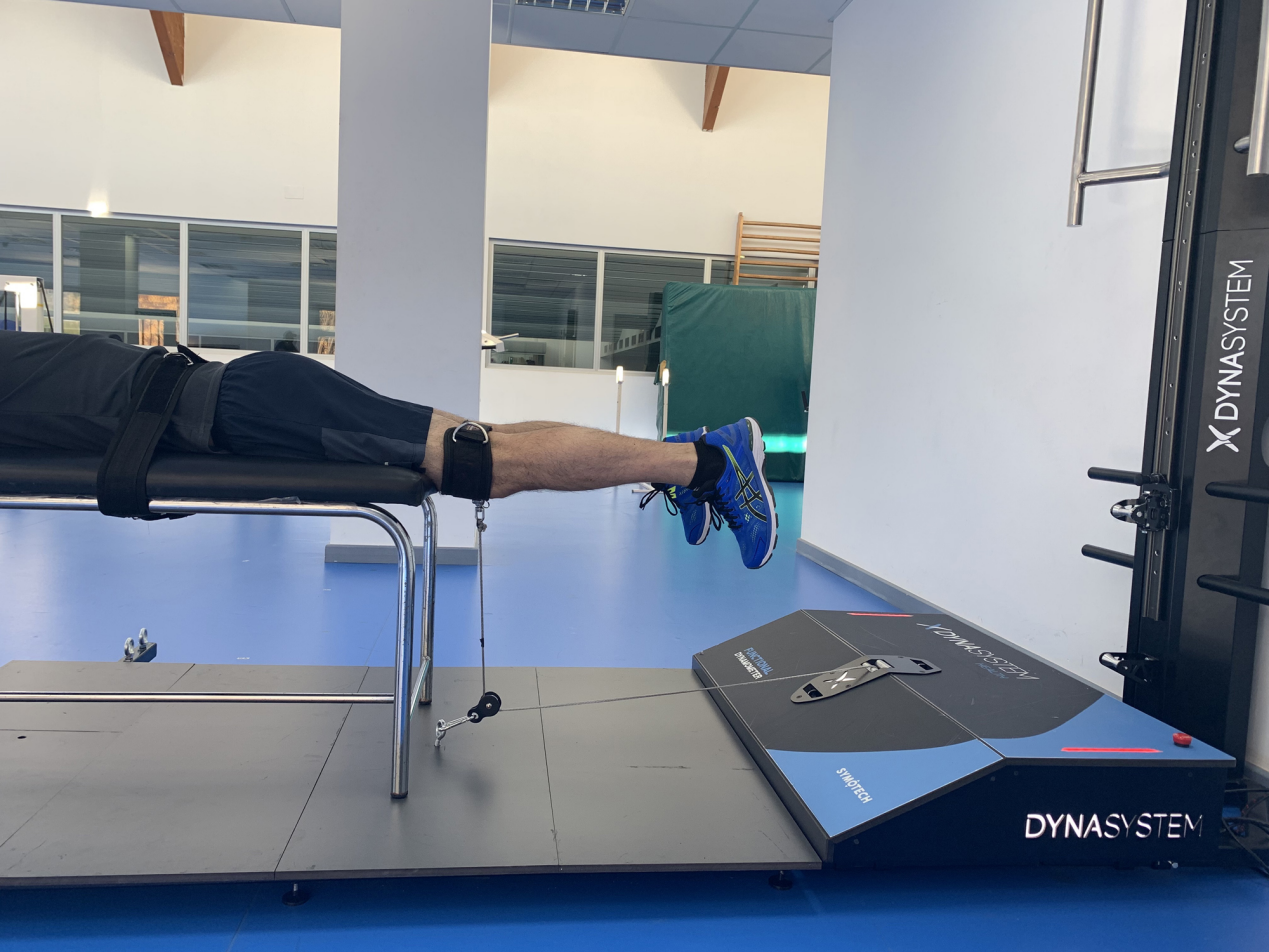
Hip extension. Isometric hip extension using a functional electromechanical dynamometer.

Hip extension was measured with the subject in prone position on a stretcher, with knees extended outside the stretcher at an angle of 0°. In this position, the subject was instructed to perform a hip extension muscle contraction as strong as possible against a fixed resistance provided by the DEMF (Fig. 2).

### Hip abduction

**Figure 3:**
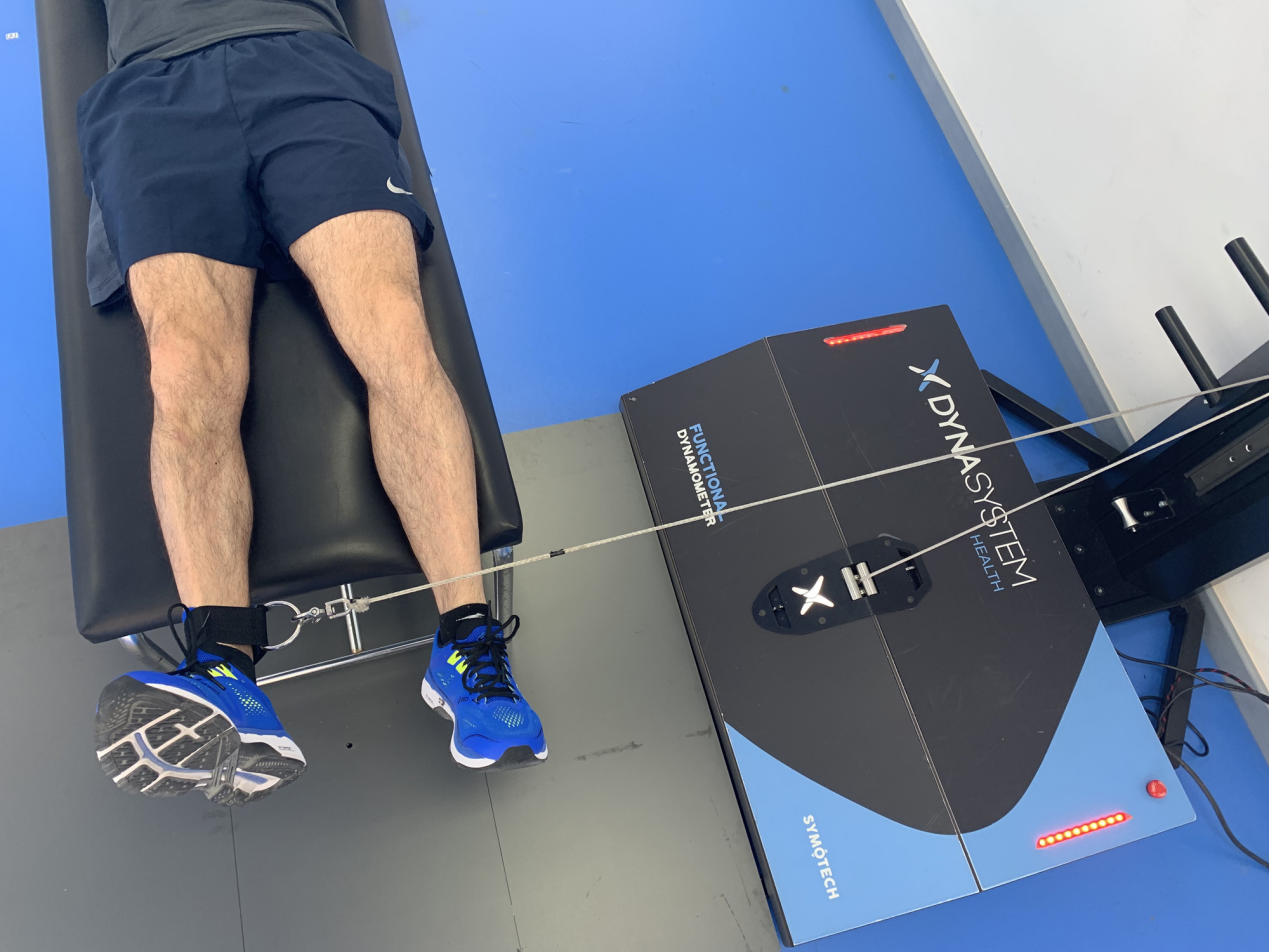
Hip abduction. Isometric hip abduction using a functional electromechanical dynamometer.

Hip abduction was measured with the subject lying prone on a stretcher with legs in a neutral position. The axis of the hip joint was perpendicular to the axis of the DEMF pull. The ankle was firmly fixed with a strap attached to the pulley of the device. From this initial position, each subject was instructed to perform a maximum isometric contraction of hip abductors (Fig. 3).

### Hip adduction

**Figure 4:**
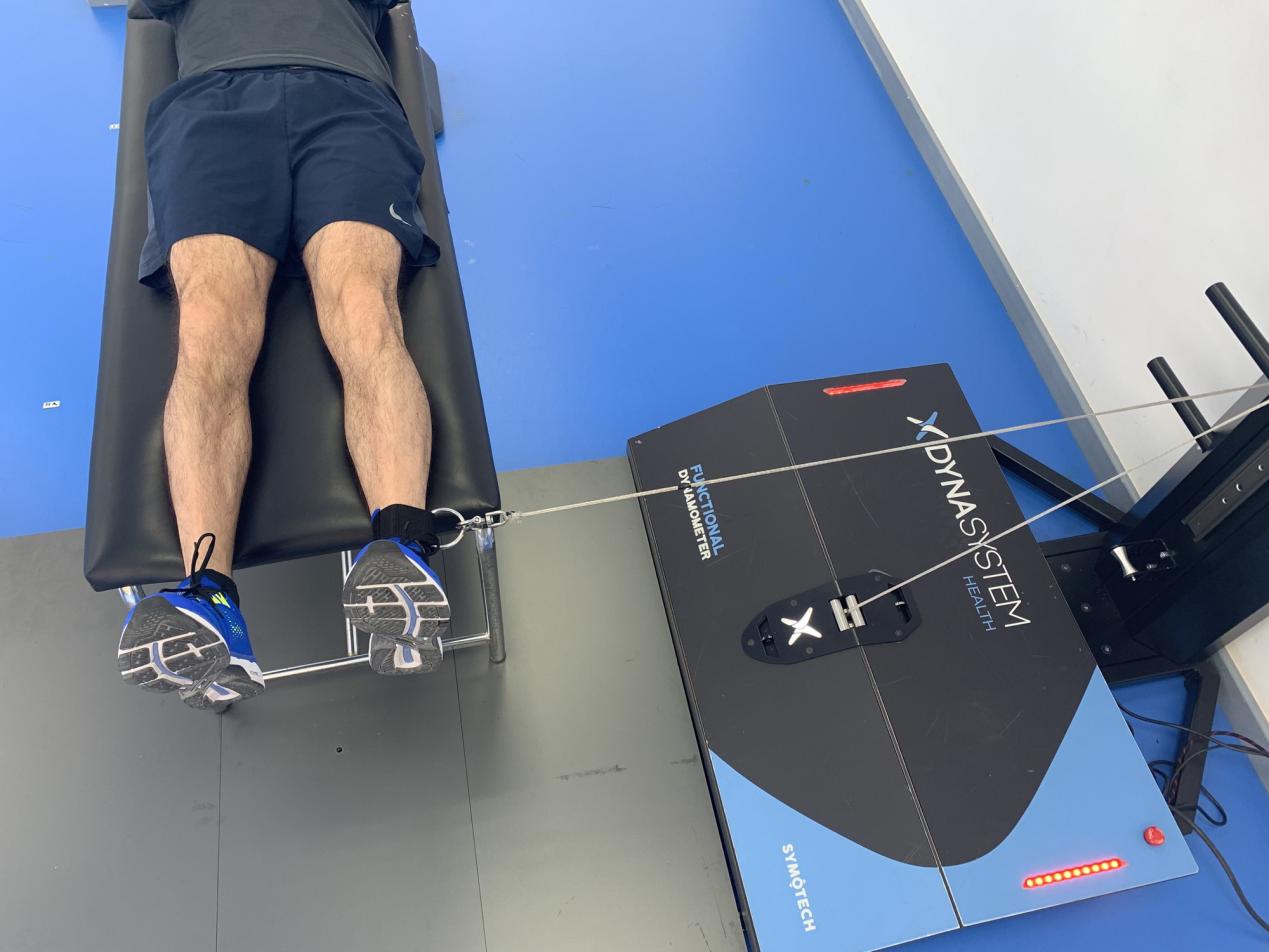
Hip adduction. Isometric hip adduction using a functional electromechanical dynamometer.

Hip adduction was also evaluated with the subject lying in prone position with the axis of the hip joint perpendicular to the axis of the DEMF pulley, but in the opposite direction. From this initial position, the subject was instructed to exert the maximum possible strength during the hip adduction (Fig. 4).

### Internal rotation

**Figure 5:**
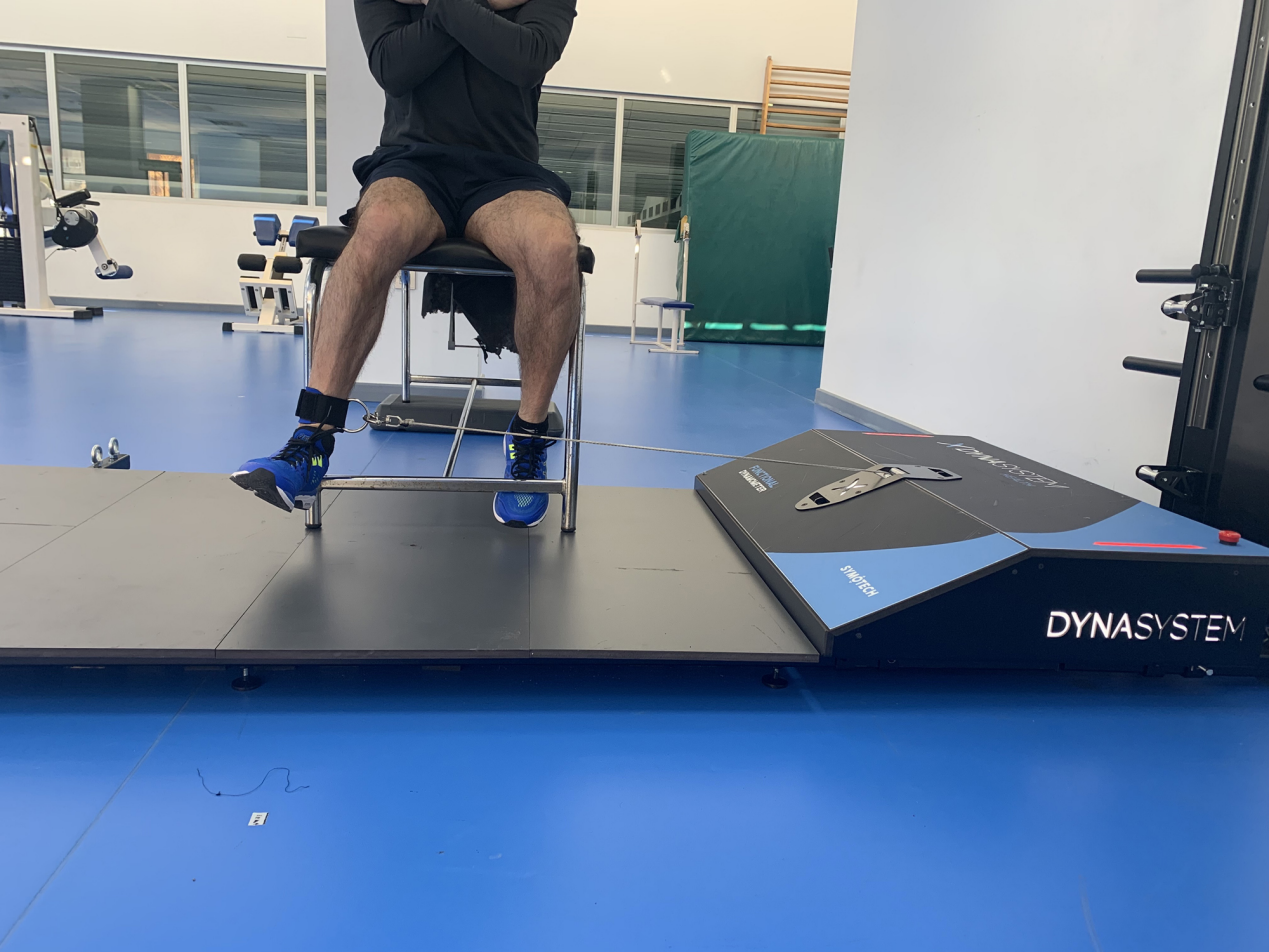
Internal rotation. Isometric hip internal rotation using a functional electromechanical dynamometer.

Internal hip rotation was evaluated with the patient sitting, so that the hip was maintained at 90° of flexion. The hip was stabilized to avoid countermoves with a belt around the knees. The axis of the hip joint was perpendicular to the axis of the DEMF pulley. The ankle was firmly attached to the pulley of the device with a strap. From this initial position, each subject was instructed to perform the maximum possible lateral strength (Fig. 5).

### External rotation

**Figure 6:**
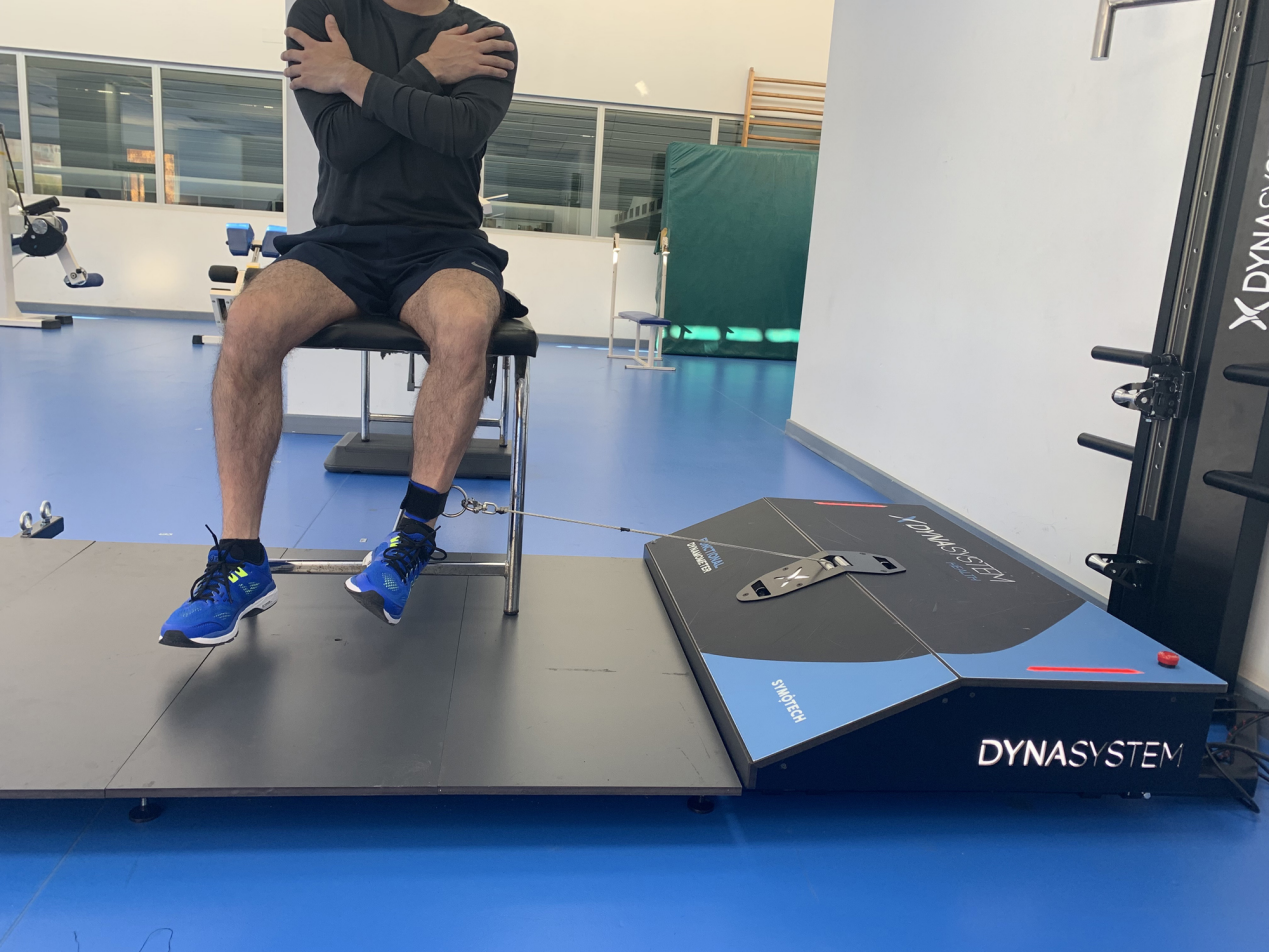
External rotation. Isometric hip external rotation using a functional electromechanical dynamometer.

External hip rotation was evaluated with the subject seated, maintaining a 90° position of hip and knee flexion. The axis of the articulation was perpendicular to the axis of the DEMF, but in the opposite direction. From this position, the subject was instructed to exert maximum lateral strength (Fig. 6).

### Sit-to-Stand Test

Subjects were asked to stand from a sitting position and then to sit ten times as fast as possible, with the arms folded across the chest so that they could not assist during the test. The test started when the examiner said “Go” and stopped when the subject got up entirely on the tenth repetition. Before the measurements, the test was performed for familiarization purposes. Measurements were performed twice with a one-minute interval of rest between the trials. The faster time was used for further analysis.

### Muscle Quality Index (MQI)

The MQI was estimated by the formula developed by Takai et al. (2009):

\begin{eqnarray*}MQI (Watts)=((Leg length\times 0.4)\times Body mass\times gravity\times 10)/Time sit to stand \end{eqnarray*}

This index included the length of the extremity expressed in meters, the height of the chair used in the sit-to-stand test (0.4 m), body mass in kilograms, gravity acceleration (9.81 ms^−2^), and a constant of 10, as proposed by Takai et al. (2009). The validity and reliability of the MQI measure have been previously reported (Barbat-Artigas et al., 2012).

### Statistical analysis

Descriptive data are presented as mean and standard deviation. Normality assumptions were tested using the Shapiro–Wilk test. A one-way analysis of variance (ANOVA) was used to compare differences in MQI, time sit-to-stand and peak force between groups. A Scheffé multiple comparison test was used after the ANOVA. Pearson correlation coefficients were calculated to examine associations between MQI and peak force of hip muscles and body composition. The coefficient of correlation was interpreted through classifications described by Mukaka (2012), where 0.9 to 1.0 was very high correlation, 0.7 to 0.9 was high, 0.5 to 0.7 was moderate, 0.3 to 0.5 was low, and 0.0 to 0.3 was negligible correlation. Statistical significance was set at *p* ≤ 0.05. Statistical analyses were performed using STATA version 15.0.

## Results

The MQI is lower in subjects with hip osteoarthritis, with no significant differences between groups (*p* > 0.054). Time in the sit-to-stand test was higher in subjects with hip OA compared with healthy subjects and older adults without OA (*p* < 0.001). Subjects with OA produce less isometric strength in extension (*p* < 0.001), flexion (*p* < 0.001), abduction (*p* < 0.05), adduction (*p* < 0.001), external (*p* < 0.05), and internal rotation (*p* < 0.05) compared with healthy subjects and older adults without OA. The difference between the groups in MQI, sit-to-stand test and peak of hip strength is presented in Table 2.

**Table 2:** MQI, Sit-to-stand test and peak of hip strength in study participants.

Variables	OA Mean (SD) (*n* = 14)	HYA Mean (SD) (*n* = 32)	HOA Mean (SD) (*n* = 18)	*p* Value	OA vs HYA *p* Value	HYA vs HOA *p* Value	OA vs HOA *p* Value
MQI (W)	232.9 (81.5)	296.1 (82.3)	262.2 (75.1)	*p* 0.054	0.056	0.362	0.595
Sit-to-Stand Test (s)	12.8 (2.5)	7.8 (1.1)	10.9 (1.63)	*p* < 0.001	0.000^***^	0.000^***^	0.008^**^
PF Extension (N)	193.7 (52.9)	385.5 (107.6)	227.2 (56.7)	*p* < 0.001	0.000^***^	0.000^***^	0.553
PF Flexion (N)	278.5 (96.2)	464.9 (104.0)	324.9 (125.4)	*p* < 0.001	0.000^***^	0.000^***^	0.493
PF Abduction (N)	197.9 (40.6)	214.0 (52.9)	255.1 (70.5)	*p* 0.012	0.670	0.054	0.022^*^
PF Adduction (N)	117.8 (20.1)	143.9 (32.7)	236.4 (58.4)	*p* < 0.001	0.132	0.000^***^	0.000^***^
PF External Rotation (N)	123.5 (36.4)	177.7 (84.8)	97.2 (21.9)	*p* < 0.001	0.036^*^	0.000^***^	0.516
PF Internal Rotation (N)	159.4 (31.6)	174.7 (35.2)	300.5 (98.8)	*p* < 0.001	0.726	0.000^***^	0.000^***^

**Notes.**

MQI Muscle Quality Index OA Osteoarthritis HYA healthy young adult HOA healthy older adult SD Standard deviation

Statistical significance was established at *p* < 0.05.

* *p* < 0.05.

** *p* < 0.01.

*** *p* < 0.001.

High inverse correlation was found between MQI and sit-to-stand time (*r* =  − 0.76, *p* < 0.01) and peak force for hip abduction (*r* = 0.78, *p* < 0.01). In the OA group, moderate correlation between MQI and peak force both during flexion (*r* = 0.55, *p* < 0.05) and external rotation (*r* = 0.61, *p* < 0.05) were obtained. There was no significant correlation between body composition and MQI in subjects with OA.

Among the group of older adults without OA, an inverse correlation was found between MQI and the sit-to-stand test (*r* =  − 0.51, *p* < 0.05), peak force in flexion (*r* = 0.53, *p* < 0.05) and variables of body composition, such as percent body fat (*r* = 0.63, *p* < 0.01) and BMI (*r* = 0.59, *p* < 0.01). In healthy young adults there was a high correlation between MQI and lean mass (*r* = 0.79, *p* < 0.001), on the other hand, the association between MQI and the sit-to-stand test (*r* =  − 0.62, *p* < 0.001), peak of flexion (*r* = 0.55, *p* < 0.001) and abduction (*r* = 0.50, *p* < 0.01) force as well as with the BMI (*r* = 0.54, *p* < 0.01) is moderate and low with the peak force of adduction (*r* = 0.39, *p* < 0.05) (Table 3).

**Table 3:** Pearson’s Correlation coefficients between the muscle quality index and peak force of the hip and body composition.

Variables	OA (*n* = 14)	HYA (*n* = 32)	HOA (*n* = 18)
	Muscle Quality Index (MQI)		
Sit-to-Stand Test (s)	−0.761^**^	−0.629^***^	−0.513^*^
PF Extension (N)	0.269	0.254	0.248
PF Flexion (N)	0.552^*^	0.557^***^	0.534^*^
PF Abduction (N)	0.784^***^	0.507^**^	0.105
PF Adduction (N)	0.168	0.396^*^	0.011
PF External Rotation (N)	0.610^*^	0.085	0.420
PF Internal Rotation (N)	0.249	0.153	0.339
Body Fat (%)	0.091	−0.305	0.631^**^
BMI (kg/m2)	0.314	0.547^**^	0.594^**^
Lean Mass (kg)	−0.067	0.799^***^	0.436

**Notes.**

MQI Muscle Quality Index OA Osteoarthritis HYA healthy young adult HOA healthy older adult BMI Body Mass Index SD Standard deviation

Statistical significance was established at *p* < 0.05.

* *p* < 0.05.

** *p* < 0.01.

*** *p* < 0.001.

## Discussion

The purpose of this study was to compare the levels of isometric strength among older adults with and without hip OA and healthy young adults, and to determine the relationship between MQI and isometric strength. The main finding of this study was that older adults with hip OA had a lower MQI and a high to moderate correlation with isometric strength levels and MQI. Our findings confirm the relation of isometric hip strength to functional capacity and MQI in older adults. These results are of clinical relevance because MQI may be a useful marker of muscle function in subjects with OA.

Regarding the first hypothesis, the MQI is lower in older adults with hip OA, but there were no significant differences between the groups of older adults and healthy young adults. This is the first study to investigate the level of MQ in subjects with hip OA. In the study developed by Brown, Harhay & Harhay (2016), which encompassed a follow-up of 14.6 years, the mean MQI corresponded to 126.2 W in subjects over 65 years, values lower than those found in our study in both older adults with OA (232.9 W) and older adults without OA (262.2 W). On the other hand, Brown, Harhay & Harhay (2016) categorized MQI into quintiles, finding that subjects who were in the lowest quintile (between 49.0–65.0) had a 57% higher probability of dying. He also found that MQI predicted mortality more accurately than the sit-to-stand test. In the study by Fragala et al. (2014), after six weeks of strength training, there was a 22% increase in MQI. This change was superior to other measures of physical function, such as gait speed, grip strength, timed chair rise, get-up and go, and read body mass. The influence of having OA of the hip, knee or both is associated with an increased risk of poor physical performance (Edwards et al., 2014). MQ could generate a diagnosis about physical performance: having a poor MQ would be influenced by fat, arthritis, pain, innervation, and decreased metabolism (Chiles Shaffer et al., 2017). In our study, the time in the execution of the sit-to-stand test is higher in subjects with OA, compared with older adults without OA and young adults. Results similar to those found by Judd et al. (2014) In which I compare subjects with OA and healthy subjects where the performance was 34% slower in the OA group in the sit-to-stand test. A longer time in the execution of the sit-to-stand implies a lower MQI, because the estimation equation includes the performance in the sit-to-stand test.

Regarding our second hypothesis, we found a high and moderate correlation of peak force at the hip with MQI and lower isometric strength in subjects with OA, compared to older adults without OA and young adults. Weakness and muscle atrophy is a characteristic present in subjects with OA regardless of severity (Loureiro, Mills & Barrett, 2013; Zacharias et al., 2016). Loureiro, Mills & Barrett (2013) found that the most significant decrease in muscle strength was in the hip and knee flexors and extensors; these results are similar to those found in our study, which show a general weakness of hip muscle strength compared to older adults without OA and young adults. In relation to the findings of Loureiro, Mills & Barrett (2013), the highest force peak in subjects with OA was in hip flexion, but these numbers were lower than those found in healthy adults without OA and young adults. In this context, positive correlations have been found between MQI and peak force in flexion (Jerez, Machado & Cerda, 2018) in obese subjects with hip OA. The flexo-extension mechanism of the hip and knee plays a fundamental role in the activities of daily life, such as sit-to-stand time from a chair (Eitzen et al., 2014; Miura et al., 2018) and walking in subjects with hip OA (Foucher et al., 2012; Hurwitz et al., 1997). Knee extensor muscle weakness was associated with an increased risk of developing knee OA in both men and women (Oiestad et al., 2015).

On the other hand, the results indicate that there is no association between body composition and MQI in the group with hip OA. The group of older adults without OA presented association between percentage of fat mass and BMI and MQI, results similar to those shown in a previous study where the MQI was only associated with a high body weight (Jerez, Machado & Cerda, 2018). These results are in contrast to this study’s findings, in which young subjects with a more significant amount of muscle mass have a higher performance in the sit-to-stand and therefore a higher MQI. It is essential to consider that high body fat was associated with lower MQ and predicts accelerated loss of lean mass (Koster et al., 2011). It has also been seen that MQ is lower in overweight and obese women (Brady et al., 2014), and a higher fibroadipose content is associated with a lower MQI (Wearing, DeBruin & Stokes, 2018).

Some aspects may be considered as limitations of our findings. In the first instance, the mechanism of detection of body composition and subsequent MQI was through bioimpedance and not, as it is generally determined, through a DXA. On the other hand, the isometric strength of knee extensors was not evaluated, despite being a mechanism that could influence the evaluation of MQI. It would also have been important to consider direct evaluation of MQ through magnetic resonance and/or ultrasound of the hip musculature; therefore, our results should be interpreted with caution and include an increase in the population studied, considering the sample size used in this study, in addition to longitudinal studies to corroborate these findings.

Despite these limitations, we consider the clinical relevance of the use of MQI as an evaluation tool in subjects with OA, as suggested by Fragala et al. (2014). MQI is a more complete muscle quality index than the determination of relative strength, since this incorporates muscle power, which is a component of neuromuscular function and evaluates specific lower extremity function related to ambulation. This is the first study that evaluates the association between MQI and isometric strength in subjects with hip OA.

## Conclusions

In conclusion, the results of this study indicate differences in MQI and isometric muscle hip strength between the subjects with OA and the group of healthy elderly and young adults. The behavior of the MQI is different in the three groups. The subjects with OA have lower MQI in addition to a high association between the isometric strength levels of the hip, the performance in the sit-to-stand test and the MQI. The MQI could be considered a tool for evaluation and follow-up in subjects with hip OA since when considering the performance of lower extremities, it would have high clinical applicability due to its accessibility and low cost of realization. Future studies are needed to determine its utility in trials including additional interventions.

##  Supplemental Information

10.7717/peerj.7471/supp-1 Data S1 Raw Data Raw measurements of the isometric strength of the hip, body composition, performance sit-to-stand test, and MQI during testing sessions.

## Additional Information and Declarations

### Data Availability

The following information was supplied regarding data availability: The raw measurements are available as a Supplemental File. The raw data shows the behaviors of muscle quality index and levels of isometric strength in the OA group, healthy older adults and young adults.
